# Aging in Place and Equity in Community Aging Care: Implications for Nursing Leadership

**DOI:** 10.1002/nop2.70726

**Published:** 2026-08-07

**Authors:** Brenda Owusu, Beatrice Marseille, Lina Paola Buitrago Ubaque, Balkys Bivins, Diana Baptiste

**Affiliations:** ^1^ University of Miami School of Nursing and Health Studies Coral Gables Florida USA; ^2^ Maple Community Care Services Inc Sydney Australia; ^3^ School of Nursing Johns Hopkins University Baltimore Maryland USA

## 1 Introduction/Background

‘Aging in place’ (AIP) refers to the capacity of older adults to continue living independently within their familiar residence and neighbourhood throughout the aging process, while having access to essential resources and assistance needed to sustain their overall well‐being (Rhodes and McNichols 2025; Szanton and Bonner 2022; Wegener et al. 2023). Aging in place is now a key focus in many health systems and communities in the United States, incorporated into community and home‐based care programmes.

Community‐dwelling older adults can be supported through different approaches in the United States; programmes such as the Community Advancing Better Living for Elders (CAPABLE) and the Rebuilding Together Richmond (RT‐R) support AIP efforts to improve equitable access to community and home‐based care (Rhodes and McNichols 2025; Szanton and Bonner 2022). These nurse‐led, person‐centred home‐based programmes employ multidisciplinary approaches targeted towards improving medication management, preserving physical strength, nutrition and home safety while reducing isolation, depression and fall risks among older adults (Rhodes and McNichols 2025; Szanton and Bonner 2022).

This paradigm shift in care supports older adults' strong desire to remain in their homes and communities as they age. Recent research consistently shows that community dwelling older adults value autonomy, familiarity and connection to social support, all of which AIP approaches can facilitate (Gonzalez‐Bautista et al. 2025; Rhodes and McNichols 2025; Szanton and Bonner 2022; World Health Organization 2024). In response, health systems and communities have expanded home‐based care, digital health monitoring and interventions, and supportive community services (Gonzalez‐Bautista et al. 2025; Wegener et al. 2023). However, the opportunity to age safely at home is unequally accessible for older adults managing chronic conditions, cognitive or functional limitations, limited digital access or socioeconomic disadvantage; frequently encounter significant obstacles in accessing coordinated preventive care and culturally appropriate community resources (Gonzalez‐Bautista et al. 2025; Rhodes and McNichols 2025; Szanton and Bonner 2022; Wegener et al. 2023). Emerging evidence suggests that when gaps in digital access and technology support are intentionally addressed, aging‐in‐place outcomes may improve, underscoring digital access and usability of these technologies as an important component of equitable community aging strategies (Gonzalez‐Bautista et al. 2025; Wegener et al. 2023).

### 1.1 Equity Gap in Aging in Place

Despite broad policy support in the U.S., AIP initiatives are frequently implemented through fragmented medical, social and community programmes with limited integration across sectors. This limitation creates stark disparities in who can access transportation, housing modifications, navigation support and ongoing clinical management (Gonzalez‐Bautista et al. 2025; Rhodes and McNichols 2025; Szanton and Bonner 2022). These gaps typically fall along familiar lines of inequalities, social and structural determinants of health, leaving marginalized populations at persistent disadvantage. As a result, AIP programmes may function well for some older adults with strong support systems while amplifying risk and care burden for others. In practice, this means aging in place can operate as a benefit for the well‐supported and a risk for those who are under‐supported (Gonzalez‐Bautista et al. 2025; Rhodes and McNichols 2025; Szanton and Bonner 2022; Wegener et al. 2023). Bridging these divides will require intentional equity‐centred frameworks that promote coordination and embedding nursing expertise into community‐based aging care.

**FIGURE 1:**
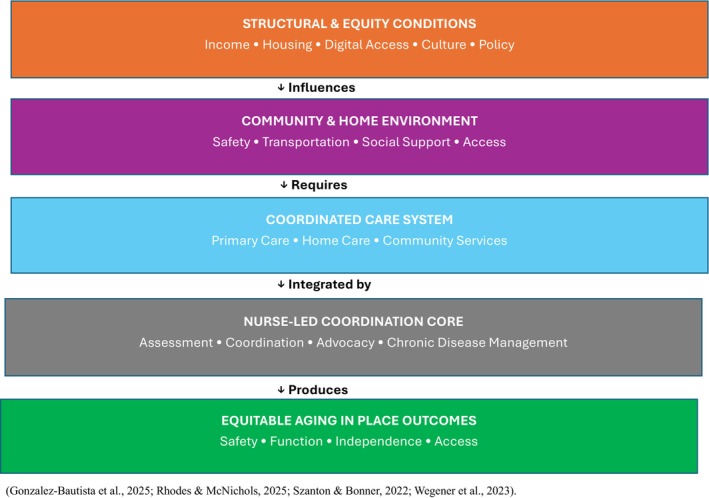
Equity‐Centered ‘Aging in Place’ Approaches (Gonzalez‐Bautista et al. 2025; Rhodes and McNichols 2025; Szanton and Bonner 2022; Wegener et al. 2023).

Equity‐centred AIP approaches can be achieved by addressing the structural conditions, community environment and coordinated care layers integrated through nurse‐led coordination to achieve safe and equitable community aging (Figure 1). This proposed model depicts the characteristics in a stepwise approach for achieving equitable aging in place outcomes. At the top level, structural and equity conditions, including income, housing, digital access, culture and policy influence community and home environments, which encompass safety, transportation, social support and access (Gonzalez‐Bautista et al. 2025; Rhodes and McNichols 2025; Szanton and Bonner 2022; Wegener et al. 2023). These environmental factors require a coordinated care system comprising primary care, home care and community services. This system can be integrated by a nurse‐led coordination core responsible for assessment, coordination, advocacy and chronic care management. Together, these interconnected layers ultimately produce equitable AIP outcomes characterized by safety, function, independence and access for older adults (Gonzalez‐Bautista et al. 2025; Rhodes and McNichols 2025; Szanton and Bonner 2022).

Recent global policy guidance makes clear that transforming care for older adults requires structural and equity‐focused redesign, not simply expansion of existing services (Gonzalez‐Bautista et al. 2025; World Health Organization 2024). Without explicit equity targeting, AIP strategies risk becoming aspirational for some and unsafe for others. Equity must be built into AIP models by design, not evaluated only after implementation. Programmes like CAPABLE and RT‐R demonstrate that dignity, functional independence and safety can be operationalized through structured, home‐based interventions for older adults (Rhodes and McNichols 2025; Szanton and Bonner 2022).

### 1.2 Why Nurses Must Lead

Nurses are uniquely positioned to lead equitable AIP redesign because nursing practice sits at the intersection of clinical management, functional assessment, care coordination and community engagement. National nursing policy guidance has explicitly called for expanded nursing leadership and system redesign roles to advance health equity, including in community‐based and aging care settings (Flaubert et al. 2021). Across primary care, home health, public health and advanced practice roles, nurses routinely integrate medical and social information to guide person‐centred care for older adults with complex needs. This cross‐setting presence enables nurses to identify risk early, coordinate services, support self‐management of chronic conditions and address barriers related to access and health literacy (Flaubert et al. 2021; Gonzalez‐Bautista et al. 2025; World Health Organization 2024). International calls to transform care systems for older adults emphasize integrated, person‐centred approaches, areas in which nursing practice already has deep operational experience (World Health Organization 2024). Placing nursing leadership at the centre of aging‐in‐place models shifts care from fragmented delivery to coordinated, equity‐oriented community practice.

### 1.3 Implications for Nursing

Equitable aging‐in‐place models require nursing roles that extend beyond episodic clinical care to longitudinal coordination and risk management in community settings. Nurses and advanced practice nurses should be supported to lead structured aging‐in‐place assessments that include functional status, medication safety, home environment and social and digital access barriers (Rhodes and McNichols 2025; Szanton and Bonner 2022). Nursing practice can operationalize equity by embedding vulnerability screening, care navigation and culturally responsive education into home and primary care encounters (Rhodes and McNichols 2025; Szanton and Bonner 2022; Wegener et al. 2023). Expanded nurse‐led models in primary care, home health and community programmes can improve early risk identification and reduce preventable decline (Rhodes and McNichols 2025; Szanton and Bonner 2022). Education programmes should strengthen preparation in community‐based geriatric services, equity‐focused assessment and cross‐sector coordination (Gonzalez‐Bautista et al. 2025). At a systems level, reimbursement and programme design should explicitly include nurse‐led coordination functions. Positioning nursing in accountable coordination roles shifts aging in place from a service goal to a managed care model.

## 2 Conclusion/Call to Action

The future of aging in place will be determined by policy endorsement that supports accountability for coordination across agencies and equity in practice. That responsibility should sit squarely within nursing leadership. Incremental programme expansion will not close these gaps; model redesign will. Nurse‐led, equity‐centred frameworks must become the standard architecture for community aging care, not pilot exceptions. This requires investment in advanced practice nursing roles, cross‐sector care coordination and equity‐based outcome measurement. The question is no longer whether aging in place is desirable, but whether it is being built safely and fairly. Nursing has the workforce reach and clinical perspective to lead this transformation and should be both empowered and expected to do so.

## Author Contributions

**Beatrice Marseille:** writing – original draft. **Brenda Owusu:** conceptualization, writing – original draft, writing – review and editing, supervision. **Lina Paola Buitrago Ubaque:** writing – original draft. **Diana Baptiste:** writing – original draft, supervision, writing – review and editing. **Balkys Bivins:** writing – original draft, writing – review and editing, supervision.

## Funding

The authors have nothing to report.

## Conflicts of Interest

The authors declare no conflicts of interest.

## Data Availability

Data sharing not applicable to this article as no datasets were generated or analysed during the current study.
